# Improving Long-Range Intramolecular Proton Transfer—Further Molecular Design Using the Successful Molecular Switch 8-(Benzo[d]thiazol-2-yl)quinolin-7-ol (HQBT) as a Structural Model

**DOI:** 10.3390/mi17091084

**Published:** 2026-09-15

**Authors:** Daniela Nedeltcheva-Antonova, Nikoleta Kircheva, Silvia Angelova, Liudmil Antonov

**Affiliations:** 1 Institute of Electronics, Bulgarian Academy of Sciences, 1784 Sofia, Bulgariankircheva@iomt.bas.bg (N.K.); 2 Institute of Organic Chemistry with Centre of Phytochemistry, Bulgarian Academy of Sciences, 1113 Sofia, Bulgaria; 3 Institute of Optical Materials and Technologies “Acad. J. Malinowski”, Bulgarian Academy of Sciences, 1113 Sofia, Bulgaria; sea@iomt.bas.bg; 4 University Centre on Tautomeric Research and Education in Science and Technology (ERA Chair UCTREST), University of Plovdiv, 4000 Plovdiv, Bulgaria

**Keywords:** molecular switch, proton crane, tautomerism, proton transfer, quinoline-7-ol, hydroxy benzodiazines, DFT

## Abstract

Proton cranes are single-molecule photoswitches with rotor and stator parts attached, where the switching event is based on a multi-step, long-range intramolecular proton transfer within the stator. The process of intramolecular motion makes such structures prototypes for machines at a nanomolecular level with broad potential applications as novel materials, necessitating a multifaceted approach to their investigation. Theoretical design of conjugated tautomeric proton cranes, using benzothiazole as a tautomeric rotor and a variety of tautomeric OH-containing heterocycles as possible stators, has been attempted by using density functional theory calculations in various environments. The shape of the ground-state potential energy surface has been used to estimate the suitability of possible proton cranes. A previously developed and studied proton crane, named **HQBT**, in which the benzothiazole rotor is attached to the eighth position of the quinoline-7-ol stator, has been used as a comparative example. The results indicate that under certain conditions, cinnolin-7-ol-based proton cranes could have practical applicability in non-polar media, where the performance of **HQBT** is not satisfactory.

## 1. Introduction

Long-range intramolecular proton transfer (LRIPT) is a process of exchanging a proton over a long distance within the same molecule. The major difference between it and short-range IPTs [1] is the lack of intramolecular hydrogen bonding, which, by connecting the proton donor (PD) and proton acceptor (PA) parts of the molecule, serves as an axis for a single-step proton exchange. Both processes can be either tautomeric, achieved by the rearrangement of the double bonds between the PD and PA, or zwitterionic.

**Scheme 1:**
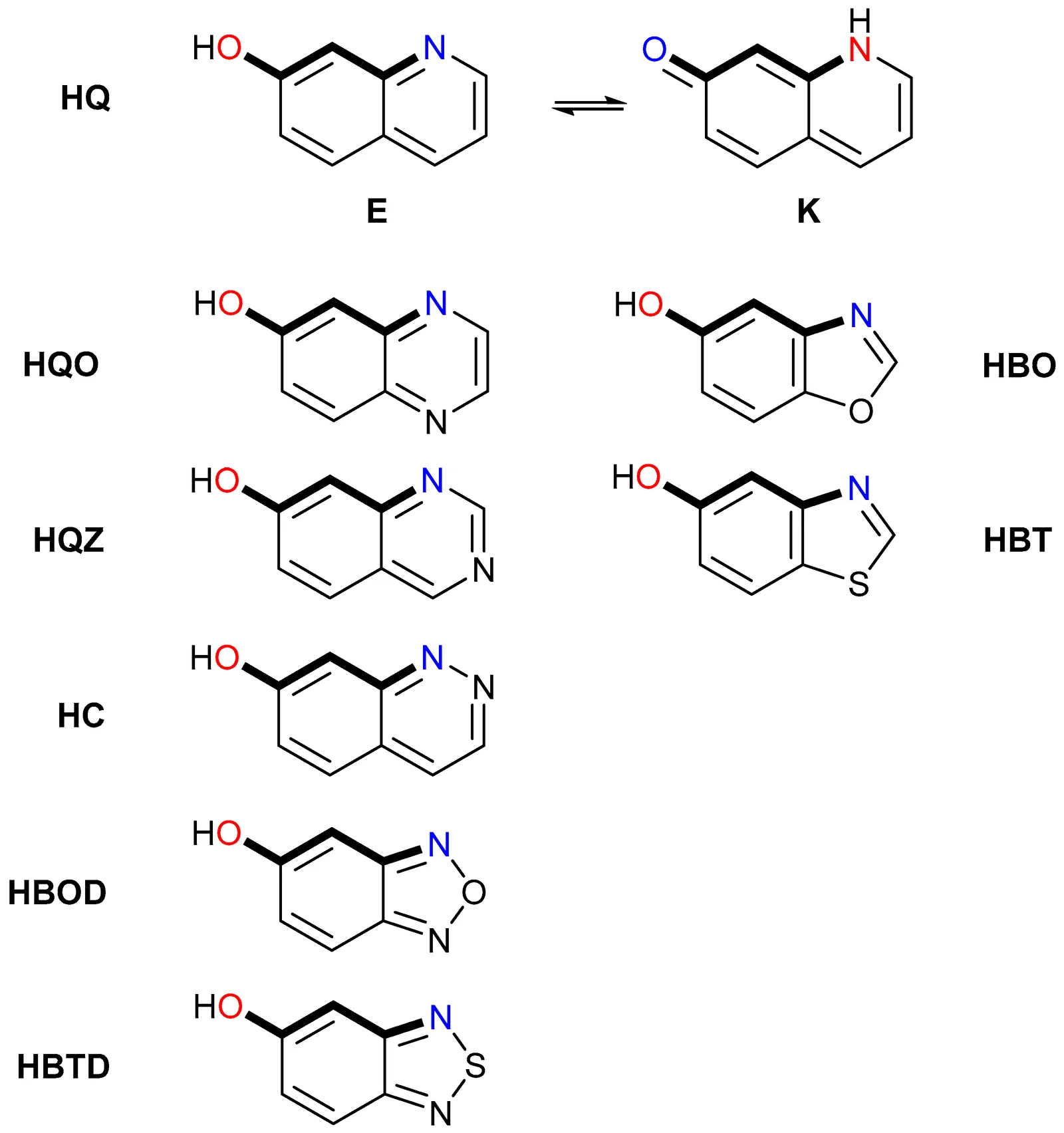
Systems with potentially possible LRIPTs presented in their **E** forms: **HQ**—quinoline-7-ol, **HQO**—quinoxalin-6-ol, **HQZ**—quinazolin-7-ol, **HC**—cinnolin-7-ol, **HBOD**—benzo[c][1,2,5]oxadiazol-5-ol, **HBTD**—benzo[c][1,2,5]thiadiazol-5-ol, and **HBO**—benzo[d]oxazol-5-ol, **HBT**—benzo[d]thiazol-5-ol. The LRIPT backbone is marked in bold. The tautomeric equilibrium in **HQ** is shown as an example at the top. In the left column, LRIPT leads to a tautomeric structure, while in the right, it results in a zwitterionic NH structure. The PDs are given in red, while PAs are colored in blue.

The large distance between the PD and PA makes LRIPT a multi-step process with a complicated mechanism. Quinolin-7-ol (**HQ**, Scheme 1), one of the most studied LRIPT systems [2,3,4,5,6,7,8,9,10,11,12], is a typical example. Here, the OH group (PD) and the N atom (PA) are far apart to provide conditions for a direct, truly intramolecular proton exchange. The enol tautomer (**E**) is substantially more stable, while the NH tautomer (**K**) has been experimentally observed only in protic organic solvents or in the presence of water or ammonia, and is the result of an intermolecular, multi-step, solvent-assisted proton exchange [12,13]. In non-polar solvents, the formation of cyclic dimers provides an additional opportunity for concentration-assisted proton exchange [14,15,16,17,18,19]. Although the exchange of the proton in both cases occurs within the same molecule as an overall result, the real mechanism gives a good reason to classify the process as pseudo-intramolecular PT [20].

**Scheme 2:**
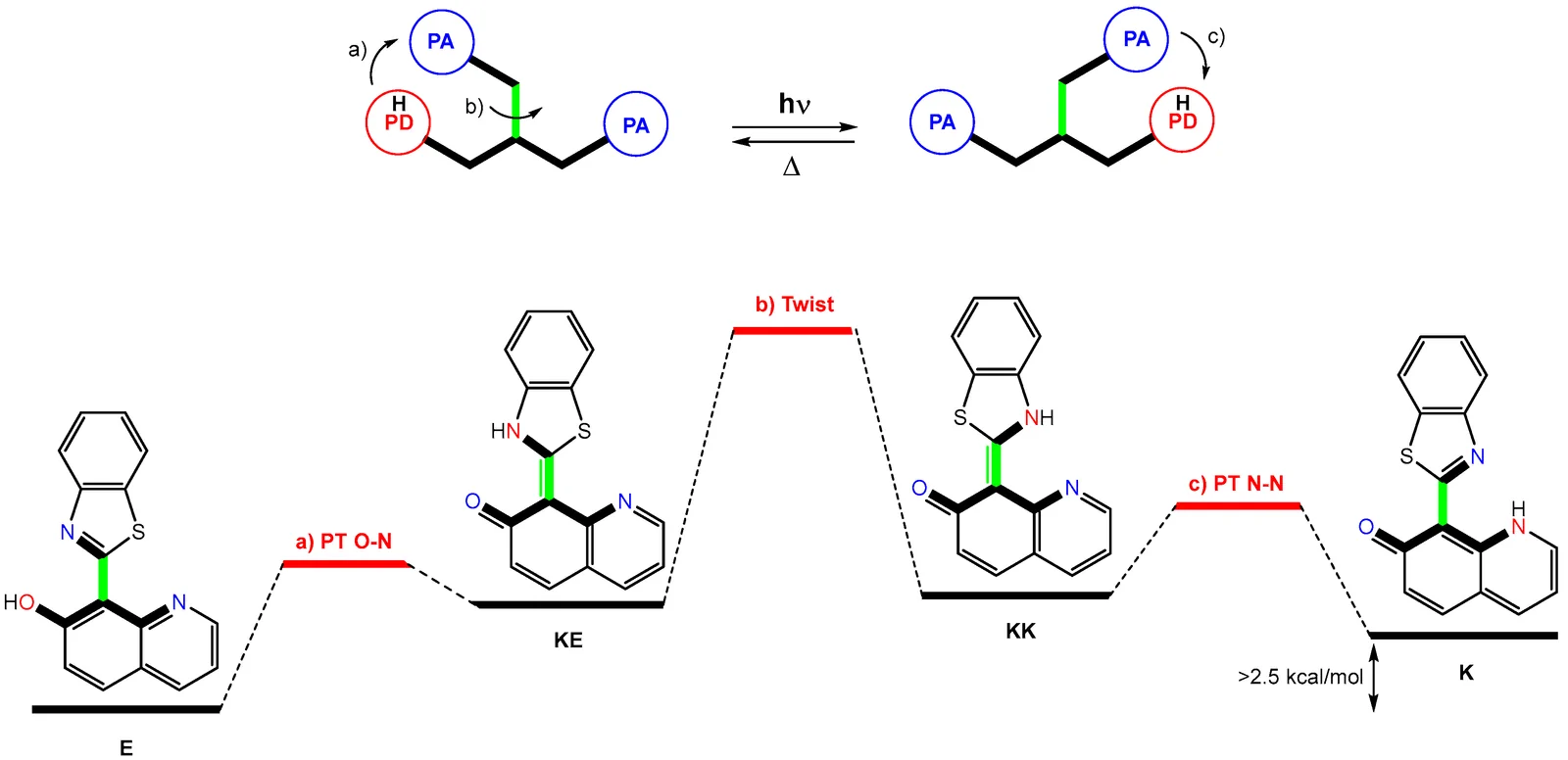
Conceptual sketch of the action of a proton crane upon irradiation and the potential energy surface in the ground state of **HQBT**. The PD sites are colored red, while the PAs are given in blue. The green bond highlights the central C–C (or C=N) linkage undergoing conformational changes along the reaction coordinate; the curved arrow indicates the direction of rotation about this bond during the twist step (**b**); dashed lines connect the optimized ground-state structures to their relative energy levels on the diagram.

A true LRIPT can only be achieved by a structural modification, as shown in Scheme 2. In this case, the implemented sidearm (rotor) transfers the proton, like cargo, over a long distance, which naturally leads to the name “proton crane” [21]. The overall mechanism includes an initial PT (a), in which the proton is captured by the rotor, followed by twisting of the protonated rotor (b), which delivers and releases the proton via a second PT step (c). The intramolecular rotation of the rotor, with respect to the stator, makes such compounds prototypes of micromachines at the nanomolecular level operating under a variety of stimuli [21]. With few exceptions [22,23], most of the existing proton cranes are based on **HQ** as a stator [13,20,24,25,26,27,28,29,30,31,32]. Recently, we designed a highly efficient proton crane in which **HQ** is used as a stator and a benzothiazole unit (**BT**) is attached at the eighth position as a rotor. As has been experimentally proven, upon irradiation, this compound (8-(benzo[d]thiazol-2-yl)quinolin-7-ol, **HQBT**) acts as a highly efficient proton crane [29]. Clean switching from **E** to **K** has been reported in acetonitrile. Based on the experimental data, theoretical modeling was performed to increase its efficiency, either through structural modifications of the rotor [32] or through suitable substitution of the **HQ** stator [31]. As a result, conditions regarding the shape of the potential energy surface (PES) in the ground state were defined to guide the molecular design of proton cranes.

As mentioned above, most of the existing proton cranes are based on the use of **HQ** as a stator. Therefore, in the current communication, we theoretically investigate how replacing **HQ** with other heterocycles (as listed in Scheme 1) in the **BT**-containing proton cranes that are structurally capable of exhibiting LRIPT would affect the ground-state PES. The shape of the PES will be used as a first step in screening their suitability for the design of new, promising proton cranes.

## 2. Theoretical Methodology

Quantum chemical calculations were performed using the Gaussian 16 C.01 program suite [33]. All structures were optimized without restrictions, using tight optimization criteria and an ultrafine grid in the computation of two-electron integrals and their derivatives. The true minima were verified by performing frequency calculations in the corresponding environment. Implicit solvation was described using the Polarizable Continuum Model [34] (the integral equation formalism variant, IEFPCM, as implemented in Gaussian 16). Transition states were localized using the STQN method [35] and verified by performing frequency calculations in the corresponding environment. Natural Bond Orbital analysis was performed using NBO version 3 [36], as implemented in Gaussian 16.

The M06-2X [37,38] functional with the TZVP [39] basis set was used for structure optimizations in the ground state. The use of M06-2X provides very good predictability of the ground-state [28,29,40,41,42] tautomeric composition in tautomeric compounds and proton cranes in solution, as well as the E/Z isomerization ratio in some rotary switches. The Harmonic Oscillator Model of Aromaticity (HOMA) indices [43,44,45,46], based on the M06-2X optimized ground-state geometries, were calculated by using the Multiwfn software package (version 3.8(dev)) [47,48].

In addition, the DFT results for the PES of **HQBT** were previously validated [29] by using domain-based local-pair natural orbital-coupled cluster singles and doubles and perturbative triple excitations (DLPNO-CCSD(T)) [49].

Bearing in mind that M06-2X systematically underestimates the absorption band positions [50], the UV–Vis spectral data were predicted by using the B3LYP functional with the basis set as above in the corresponding environment using the M06-2X-optimized geometries. The spectra were simulated according to the methodology described previously by us [51] by using a single Gaussian band shape with a half-bandwidth (${\Delta \nu }_{1/2}$) of 3000 cm^−1^. Frontier molecular orbitals (FMOs) were visualized using the Avogadro software package (version 1.2.0).

## 3. Results and Discussion

The tautomerism of **HQ**, as stated above, is one of the most studied cases of LRIPT in solution [2,3,4,5,6,7,8,9,10,11,12]. At room temperature, absorption (~330 nm) and emission (~380 nm) are observed from the **E** tautomer in nonprotic solvents and in DMSO [14], whereas in aqueous solution, additional fluorescence was observed (~520 nm) from the **K** form only, along with the appearance of an additional, red-shifted absorption band at ~402 nm [6]. Dual emission was also observed in alcohols [52,53,54] but with absorption only at 330 nm. This allows us to conclude that, in the ground state, the **E** tautomer is the only form present in nonprotic and nonaqueous protic solvents, whereas both tautomers are stable in nearly equal amounts in the presence of water, as estimated by Mason [4]. Solvent-assisted excited-state LRIPT was proposed based on the experimental data for protic solvents [53,55,56], where the process is facilitated by the increased excited-state acidity/basicity of the enol/imino groups [5]. The effect of the concentration also plays a substantial role [14,57]. A large number of papers have confirmed this mechanism through a variety of experimental measurements [9,10,58,59] and theoretical simulations [60,61,62,63]. Our calculations for toluene, collected in Table 1, also indicate the exclusive presence of the enol tautomer in this solvent. The predicted positions of the absorption bands are also in agreement with the values reported in the literature.

Unfortunately, only scattered data on the tautomerism of some of the compounds shown in Scheme 1 are available. The results published by Mason [4] for **HQO** and **HC** show the presence of a single enol tautomer in ethanol, with absorption bands at 350 and 355 nm, respectively, which is again in good agreement with the predictions in Table 1. In chloroform, **HQ** and **HQO** exist as enol tautomers, while the appearance of a strong C=O peak was found for **HC** in the solid state by using IR spectroscopy [3]. At pH = 5.4, both Mason [4] and Osborn [64] report the appearance of a red-shifted absorption band at 448 nm in the spectrum of **HC**, which could be attributed to the **K** tautomer. Katritzky and co-authors [65,66] demonstrated the existence of **HQZ** as an OH tautomer in DMSO using NMR. **HBTD** was studied by Angeloni in dioxane and DMSO by NMR [67], indicating the existence of only the enol tautomer. Optical spectral data for its OMe derivative in methanol and toluene show absorption bands at 337 and 338 nm [68], respectively, which can be used to validate the value of 339 nm obtained by us for the **E** form (Table 1). The corresponding emission bands appear at 421 and 391 nm. Spectral and tautomeric properties of the remaining compounds have not been studied before.

The relative energies collected in Table 1 indicate strong stabilization of the **E** tautomer in toluene compared with the keto form. Compared with **HQ**, the introduction of a second heteroatom leads either to destabilization of the **K** tautomer, as in **HQO**, or to stabilization, as predicted for **HQZ** and **HC**. The strong electron-acceptor character of the heterocyclic core in **HBOD**/**HBTD** further destabilizes the keto form, a tendency that is very pronounced in **HBO**/**HBT**, where proton transfer leads to strongly polar zwitterionic structures. The transfer from toluene to acetonitrile leads to substantial stabilization of the more polar **K** tautomer, while the relative trends across the compounds remain unchanged.

The relative stabilization of the tautomers can be regarded as a function of the relative basicity of the tautomeric O and N atoms, together with the change in the aromaticity going from **E** to **K**. As a very rough approximation, pKa values can be used as an indicator of the proton-donor ability of the OH group in the enol tautomer. Such values were reported for **HQ** [5], **HQO** [69], and **HC** [64] in water, namely 8.85, 7.92, and 7.56, very similar values that correspond well to the NBO charges of the enol oxygen (Table S1). The pKa values of the corresponding nium cations are 5.48, 1.40, and 3.31, respectively, indicating the reduced basicity of the nitrogen in **HQO** compared with **HC**, which may be related, to some extent, to the reduced stability of the keto tautomer. The reported pKa values of the core heterocycles (without the OH group) [70] for **HQ** (11.96), **HQZ** (9.19), **HQO** (7.40), and **HC** (2.70) [71] do not provide a straightforward explanation for the substantial keto-form stabilization in **HC** since the NBO charges of both nitrogen atoms are very similar.

The overall charges of the individual rings, shown in Table 2, indicate a moderate charge-transfer character of the enol forms of **HQ**, **HQZ**, and **HC**, accompanied by a stronger value of opposite sign in the **K**-tautomer, which corresponds to the better thermodynamic stabilization of the latter. The charge-transfer character of the **K** forms in the remaining compounds is either very weak or absent, which could explain the higher relative energy of this tautomer. **HBT** is an exception here, in that the charge-transfer character of the keto form is strong, whereas there is no charge transfer in the enol tautomer.

Aromaticity of the individual rings in the **E** and **K** tautomeric forms was quantified using the HOMA index of Kruszewski and Krygowski [42,43], a geometry-based descriptor that compares the bond lengths within a ring to an idealized aromatic reference length. HOMA = 1 corresponds to perfect (benzene-like) aromaticity, values near 0 indicate a localized/non-aromatic ring, and negative values indicate antiaromatic character. Being purely geometric, HOMA is complementary to the NBO charges reported alongside it, separating structural (bond-length) from electronic (charge) responses to tautomerization. The compounds studied fall into three groups based on how tautomerization affects ring aromaticity.

(1) **HQ**, **HQO**, **HQZ**, and **HC** (pyridine/diazine-fused systems) show a large but incomplete loss of aromaticity in the ring bearing the phenolic oxygen upon tautomerization (OH ring → O ring), consistent with C=O bond localization upon proton transfer: HOMA falls from 0.78 to 0.83 (**E**) to 0.03–0.22 (**K**), with **HQO** (HOMA = 0.028) approaching the fully localized limit. The N ring → NH ring change is comparatively small (e.g., **HQ**: 0.87 → 0.77), indicating a more spatially localized geometric perturbation that does not propagate strongly through the fused system.
(2) **HBOD** and **HBTD** (oxadiazole/thiadiazole-fused systems) show an exaggerated version of this loss. Aromaticity is already reduced in the enol form (OH ring HOMA 0.53–0.56, well below the 0.78–0.83 range of group 1), and the keto-form O ring becomes not merely dearomatized but genuinely antiaromatic (HOMA = −0.20, −0.14). This indicates that the electron-withdrawing oxadiazole/thiadiazole substituent intensifies the quinoidal distortion at every stage of the tautomeric equilibrium rather than only upon keto-form formation.
(3) **HBO** and **HBT** (oxazole/thiazole-fused systems) show a suppressed version of this loss. Their keto-form O rings retain substantially higher aromaticity (HOMA ≈ 0.60) than any compound in groups 1 or 2, meaning the six-membered ring largely escapes the aromaticity penalty seen elsewhere. This suppression is offset by an unexpected loss of aromaticity in the enol-form N ring (HOMA 0.28–0.70), unusually low relative to the highly aromatic OH ring of the same tautomer (0.97–0.98)—a deviation not seen in groups 1 or 2, where both enol rings are comparably aromatic. Notably, this retained O-ring aromaticity in the keto form occurs despite a substantial negative NBO charge on that ring (HBT: −0.698, comparable in magnitude to the charges seen in groups 1 and 2, where such charges do coincide with low HOMA). Since HOMA and NBO charge track different properties—bond-length geometry and electron density, respectively—this decoupling is not inherently contradictory, but it does indicate that here, unlike in groups 1 and 2, charge redistribution is not matched by geometric relaxation of the six-membered ring. Instead, the structural reorganization appears to be absorbed by the five-membered azole ring, sparing the benzenoid ring from the bond-length changes that drive aromaticity loss elsewhere in the series.

In summary, ground-state enol-to-keto tautomerization is consistently accompanied by loss of ring aromaticity in the keto tautomer, which is consistent with the usual thermodynamic picture in which loss of aromaticity is one of the factors disfavoring the keto form relative to the enol in the ground state. Oxadiazole/thiadiazole fusion (**HBOD**, **HBTD**) intensifies this loss and induces antiaromaticity, while oxazole/thiazole fusion (**HBO**, **HBT**) suppresses it by redirecting the structural reorganization onto the five-membered ring, decoupling charge redistribution from six-membered-ring aromaticity loss. The present HOMA results are consistent with previous ground-state studies showing ring-selective changes in aromaticity upon tautomerization. In hydroxyquinolines, HOMA calculations show that the rings of the OH tautomers are highly aromatic (HOMA > 0.75), whereas proton transfer to the ring nitrogen can reduce the HOMA of one ring to as low as 0.1 [72], providing a close precedent for the pronounced O-ring aromaticity loss observed in Group 1. Our **HBOD**/**HBTD** compounds contain the 1,2,5-isomeric heterocycle, for which we found no direct ground-state HOMA benchmark; nevertheless, the generally reduced aromaticity reported for related oxygen-containing five-membered heterocycles—furan and oxazole (rHOMA = 0.03–0.15) and isoxazole (rHOMA = 0.32)—all classified as weakly or non-aromatic by standard Krygowski [73], and the 1,3,4-isomers of oxadiazole and thiadiazole specifically [74,75], is consistent with the pronounced aromaticity loss observed in Group 2. In benzoxazole and benzothiazole systems, HOMA is distributed unequally between the fused rings, with the aromaticity of the heterocycle depending on the nature of the heteroatom [76].

**Scheme 3:**
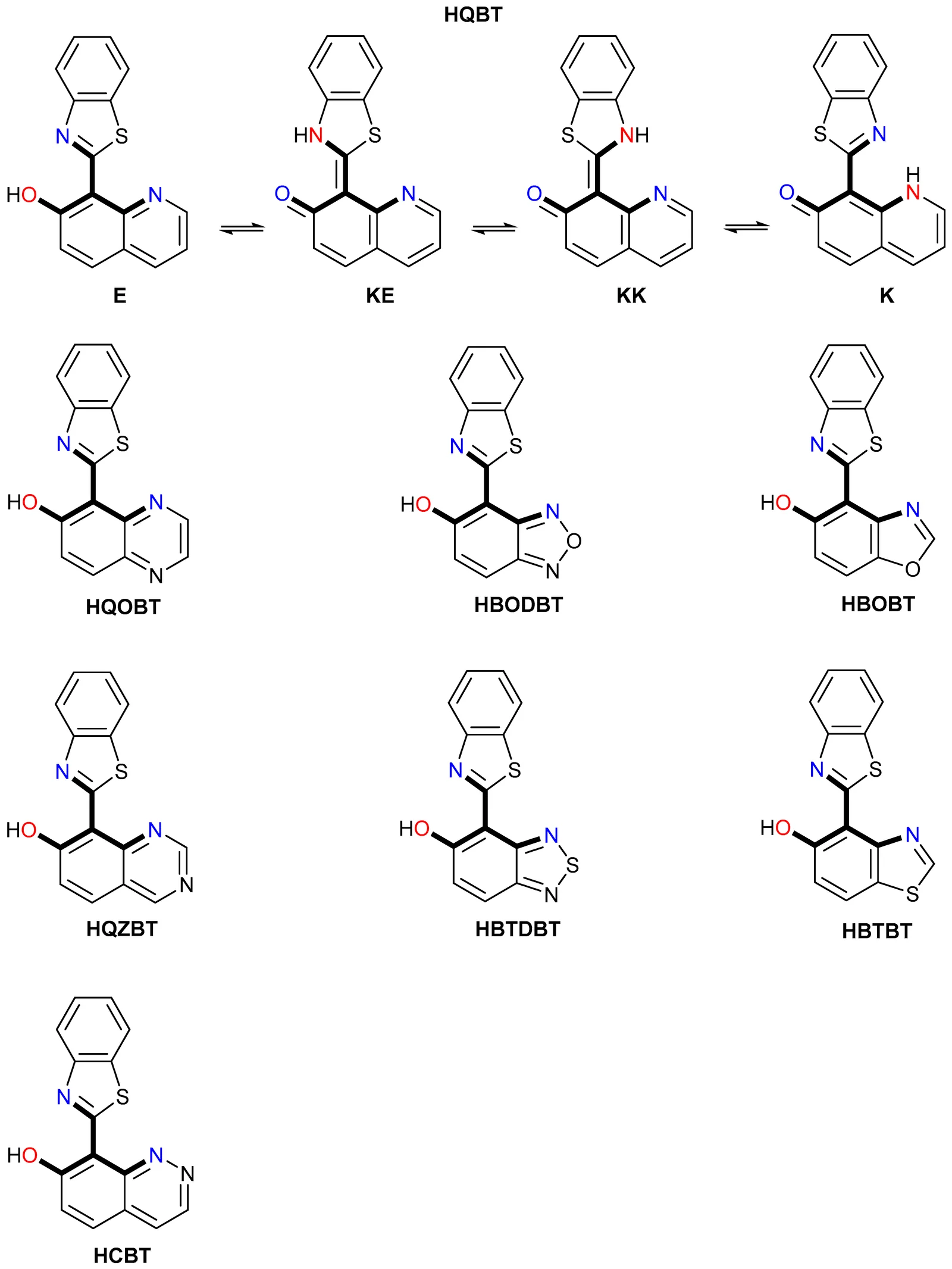
Proton cranes discussed in the current communication are shown as the initial **E** tautomer. The PT backbone is given in bold. The tautomeric transitions, leading to LRIPT, in **HQ** are shown as an example at the top.

As seen thus far, the considered heterocycles show different stabilization of the keto tautomer compared with **HQ**. It is therefore interesting to see whether the relative order is maintained when they are used as stators in proton cranes with an attached **BT** unit, following the model of **HQBT** shown in Scheme 2. In the discussion below, the abbreviations of the compounds (Scheme 3) follow the model stator-rotor naming model, taking **HQBT** as an example (the **HQ** stator is attached to a **BT** rotor, following the model sketched in Scheme 2).

Based on the known behavior of fully tautomeric conjugated proton cranes, the switching process, i.e., going from **E** to **K**, occurs in three consecutive steps, as sketched in Scheme 2. Upon irradiation, the initial **E** tautomer undergoes, in the S_1_ excited state, a very fast excited-state intramolecular proton transfer (ESIPT), giving the KE* state, in which the proton is relocated to the nitrogen atom of the rotor. As a consequence of this tautomeric process, the nature of the axle connecting the rotor and the stator changes from a single to a double bond, which fully or partially leads to twisting, resulting in the rotor and stator becoming perpendicular to each other. This point corresponds to the twisting barrier in the ground state and creates conditions under which S_0_ and S_1_ become degenerate. The resulting conical intersection region provides a channel for fast relaxation to the ground state, leading to simultaneous population of **KE** and **KK**, and eventually, under certain conditions, of **E** and **K**. Consequently, switching from **E** to **K** is achieved, after which **K** returns thermally to **E** in the ground state, at a rate determined by the ground-state twisting barrier.

This mechanism defines the requirement for the ground-state PESs of prospective proton cranes. The energies of the intermediate tautomers **KE** and **KK** must be higher than those of **E** and **K**. In this way, following excited-state relaxation of KE*, the ground-state IPT process leads to population of the end-switching forms **E** and **K**. To achieve clean switching, the energy of the initial tautomer **E** must be lower than the energies of the remaining tautomeric forms, to such an extent that the latter are not present in solution under equilibrium conditions. These requirements are very well illustrated by the PES of **HQBT**, shown in Table 3 and Figure S2. The enol tautomer is considerably more stable than the other three tautomeric forms. In toluene, the energies of **KK** and **K** are approximately the same, which, upon irradiation, leads to accumulation of a mixture of them; i.e., a switching event occurs, but not as clean switching. The situation changes in acetonitrile, where the more polar **K** is additionally stabilized, leading to essentially complete conversion of **E** to **K**, i.e., clean photoswitching [29].

**Figure 1:**
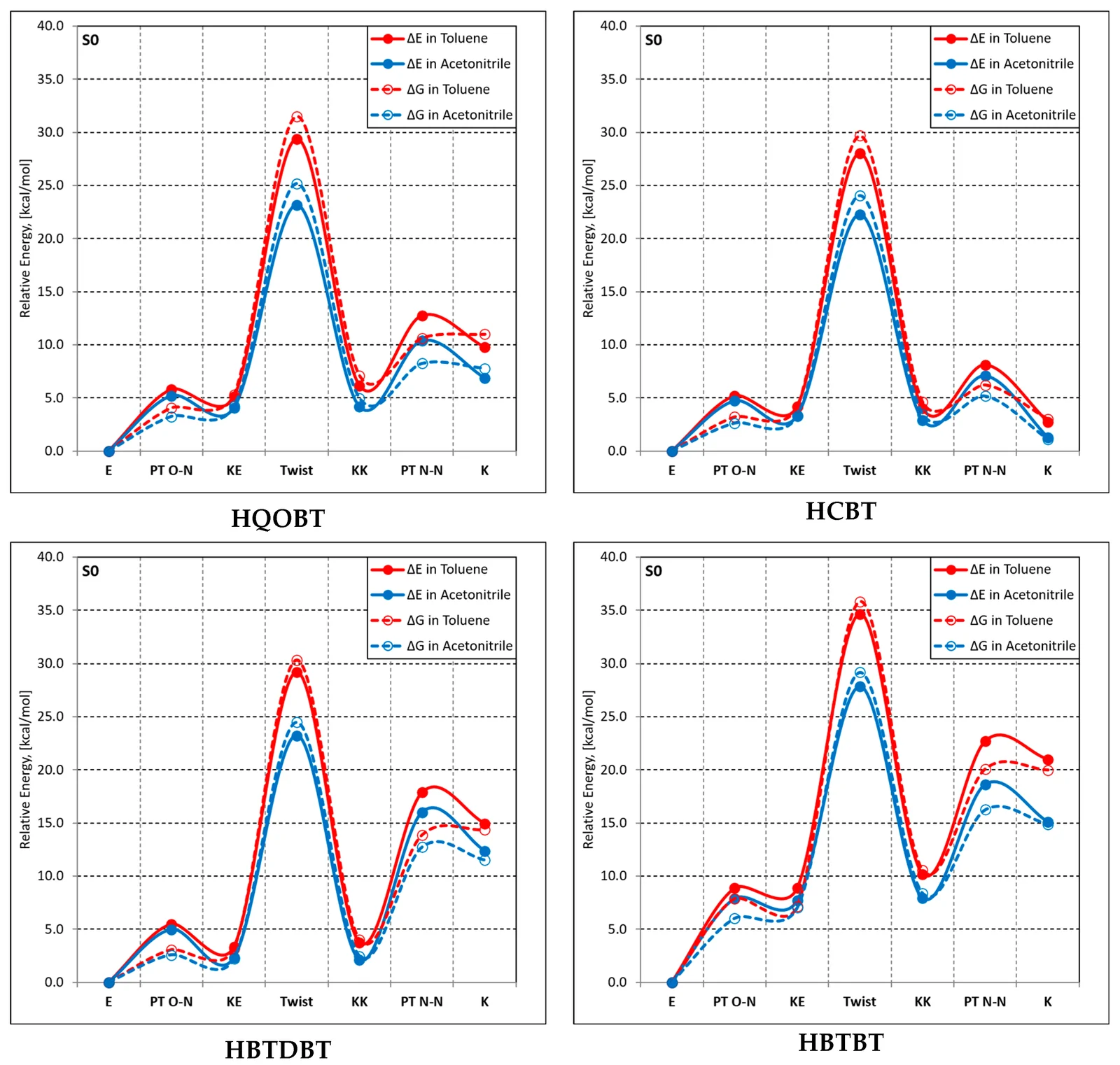
Ground-state PESs of **HQOBT**, **HCBT**, **HBTDBT**, and **HBTBT** in toluene and acetonitrile. The PESs of the rest of the compounds are shown in Figure S2. The numerical values for the relative stabilities of the tautomers are collected in Table 3.

In the case of **BT** as the rotor, the stabilization of the intermediate tautomers **KE** and **KK** is a function of the strong conjugation between the rotor and stator, and of the strength of the existing intramolecular hydrogen bonding. In both cases, the planarity of the system plays a substantial role, and it is important to note that the studied compounds, as shown in Scheme 3, are planar in all of their tautomeric forms. As seen from Table 3 and Figure 1 and Figure S2, the situation is very similar across the series **HQBT**, **HQOBT**, **HQZBT**, and **HCBT**. In all cases, **KE** and **KK** are relatively low in energy, but higher than **E**. The possibility of switching then remains a function of the relative energy of **K**. According to previous investigations, the introduction of electron-acceptor substituents in tautomeric hydroxy benzodiazines leads to stabilization of the keto tautomer [64,65]. Since **BT** is such a substituent, it could be expected that stabilization of **K** relative to **E** would be achieved, preserving the relative order of stabilization of the **K** tautomer in **HQBT**, **HQOBT**, **HQZBT**, and **HCBT** as observed in **HQ**, **HQO**, **HQZ**, and **HC**. A comparison between the data in Table 1 and Table 3 confirms exactly this.

**Figure 2:**
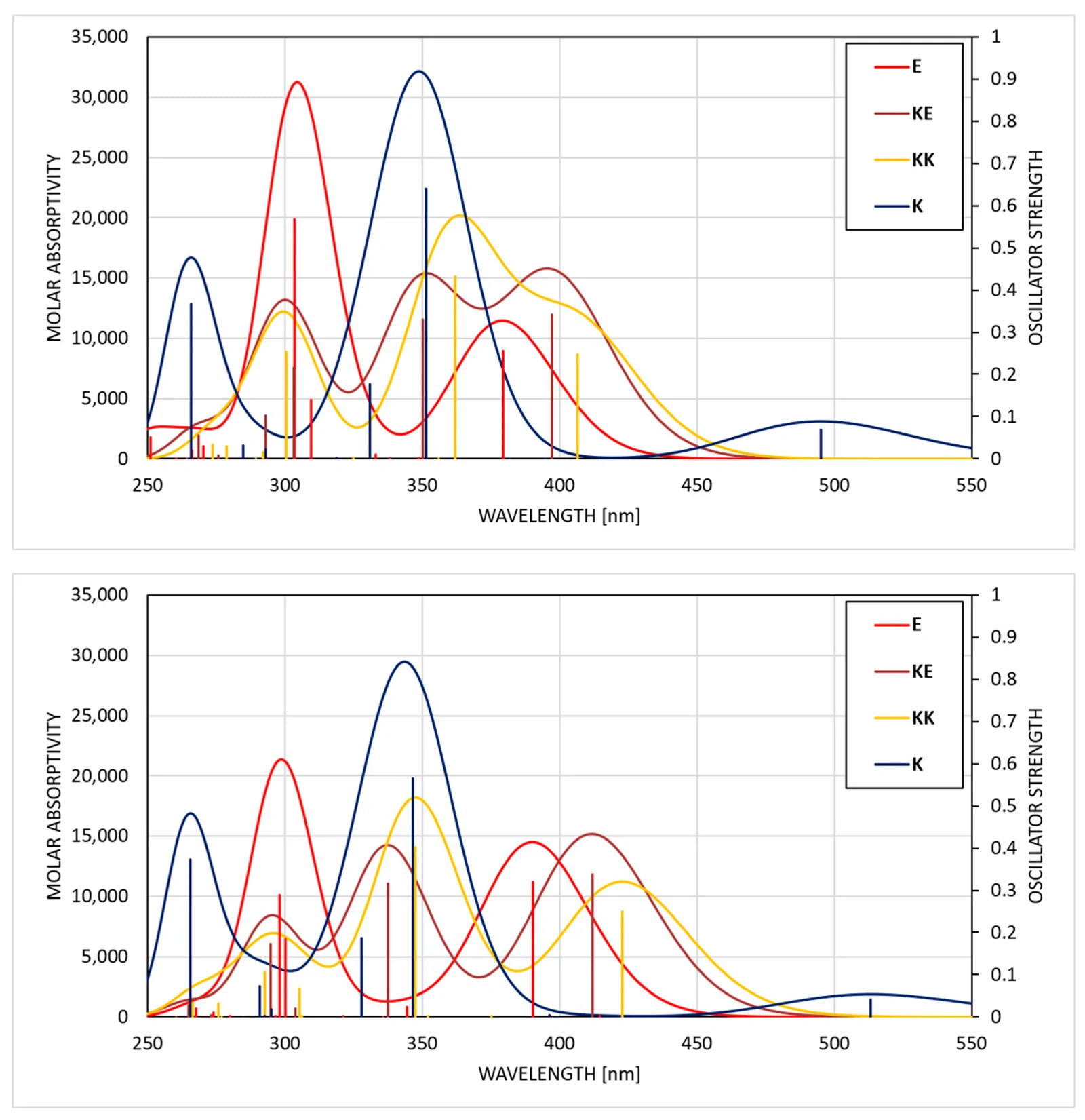
Simulated absorption spectra of (from top to bottom) **HQOBT** and **HCBT** in toluene.

However, different stabilization of **K** leads to a different picture regarding the switching process. As seen from Table 3 and Figure 1 and Figure S2, in the case of **HQOBT** the **K** tautomer is higher in energy than **KK**, which makes population of the former impossible. The situation in **HQZBT** (Figure S2) is not as drastic, but is very similar, which renders both compounds unsuitable for use as proton cranes. In these compounds, the switching process actually ends with the twisting event, without release of the proton from the rotor to the stator nitrogen atom (step c, as illustrated in Scheme 2). The stabilization of the keto tautomer in **HC** leads to a very suitable shape of the PES in **HCBT** in toluene, providing all conditions for clean switching. In acetonitrile, however, the additional solvent stabilization of the **K** form leads to a situation in which **E** and **K** could co-exist in equilibrium prior to irradiation. From the viewpoint of the expected spectral changes, irradiation should lead to the red shift in the enol maximum at ~390 nm to ~510 nm, assigned to the resulting **K**-form (Figure 2). In contrast, in **HQOBT**, no such shift could be expected because the **K** tautomer cannot be reached due to its high energy.

In **HBODBT** and **HBTDBT**, the stabilization of the end **K** tautomer is negligible compared with **HBOD** and **HBTD**, which is not sufficient to provide conditions for proton-crane action. In both compounds, the relative energy of **K** is much higher than that of the intermediate **KK**, preventing the final step of proton release. The stabilization of the **K** form in **HBOBT** and **HBTBT** is likewise insufficient for **E** to **K** switching.

**Figure 3:**
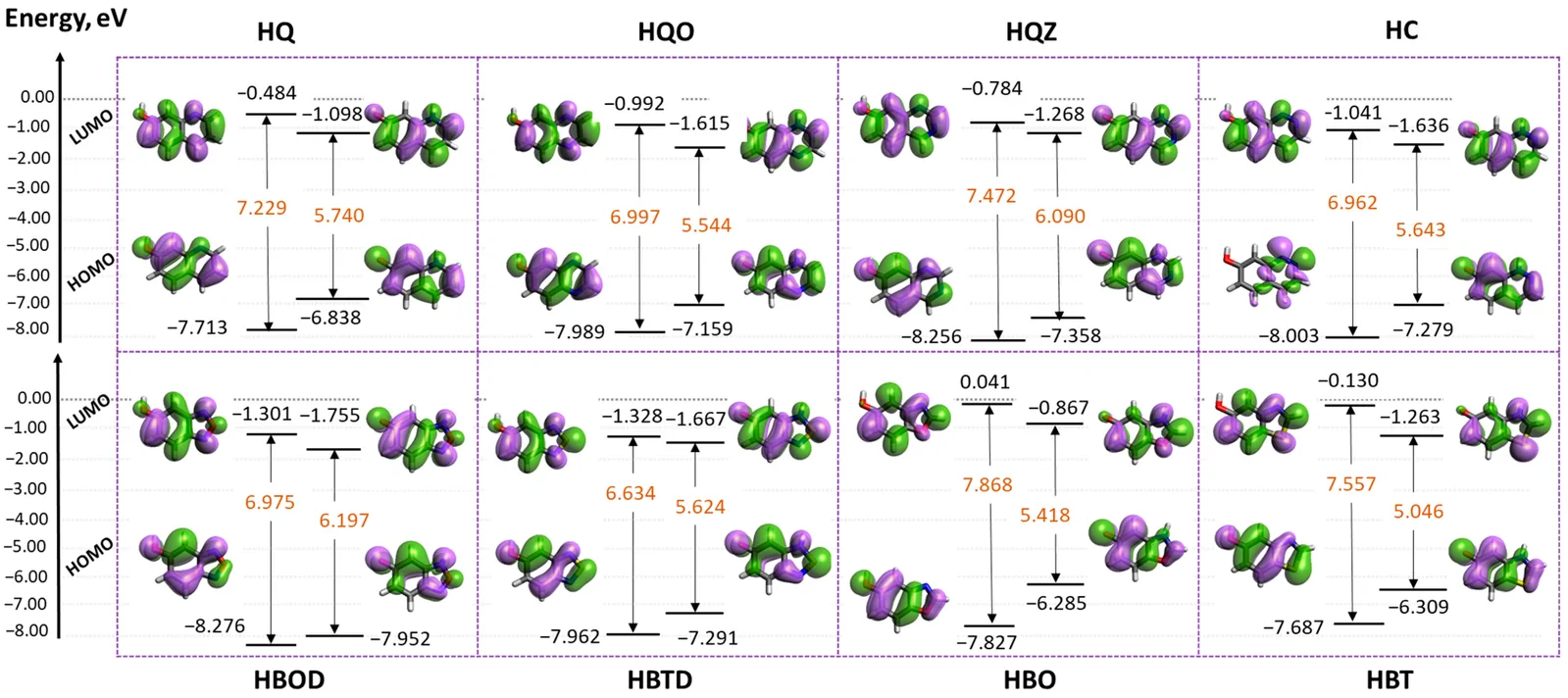
Frontier molecular orbital energy diagrams and orbital distributions for the enol (**E**, left) and keto (**K**, right) forms of the stator compounds shown in Scheme 1. The HOMO and LUMO energies and corresponding HOMO–LUMO energy gaps (ΔE, eV) are indicated for each tautomer. Purple and green isosurfaces represent opposite phases of the molecular orbitals.

**Figure 4:**
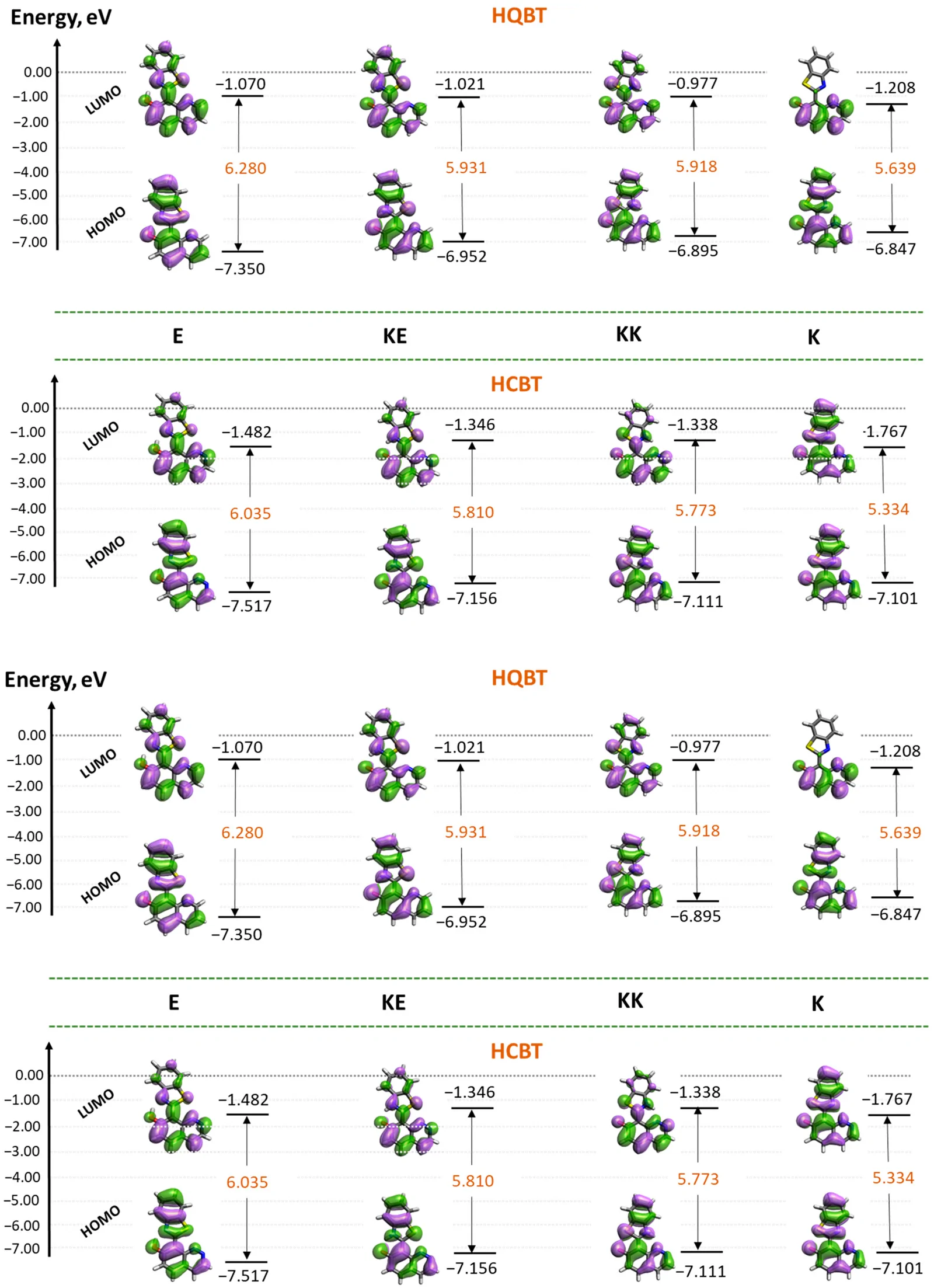
Frontier molecular orbital energy diagrams and spatial distributions of the HOMO and LUMO for the **E**, **KE**, **KK**, and **K** tautomeric forms of the proton-crane compounds **HQBT** and **HCBT**. The corresponding HOMO and LUMO energies and HOMO–LUMO gaps (ΔE, eV) are indicated for each tautomer. Purple and green isosurfaces represent opposite phases of the molecular orbitals.

Given that thermodynamic accessibility of the **K** tautomer alone does not guarantee proton-crane functionality, it is instructive to ask whether the electronic structure of these systems carries an independent signature of their switching capability. Accordingly, the vertical excitation wavelengths, oscillator strengths, and dominant orbital transitions computed at the ground-state optimized geometry of each tautomeric form are summarized in Table S2. As shown, the S_1_ (or S_2_, where S_1_ is optically dark) transition is dominated in essentially every case by a single orbital pair with a configuration interaction (CI) coefficient close to 0.70, indicating a transition of largely single-configuration characteristics throughout both the stator and proton-crane series. The frontier molecular orbitals involved in this dominant transition, namely the relevant HOMO and LUMO for each stator and proton-crane compound, evaluated at the same ground-state geometries, are presented in Figure 3 (stator units), Figure 4, and Figure S3 (selected modeled proton cranes), respectively.

The calculated vertical excitation data reveal a pronounced dependence of the lowest optically active transition on tautomeric form (Table S2). For the stator compounds, conversion from the enol to the **K** form generally produces a substantial bathochromic shift, with the dominant transitions moving from the UV region into the near-visible region. In several cases, the lowest singlet state is optically dark or nearly dark, and the first intense transition therefore corresponds to S_2_. Despite these spectral shifts, the dominant CI coefficients remain similar (ca. 0.67–0.70), indicating that the lowest relevant excitations are predominantly described by a single orbital transition.

The proton-crane compounds exhibit the same general red-shift upon proton transfer, progressing from **E** through the intermediate **KE** and **KK** forms to **K**. Their lowest bright transitions are also dominated by a single orbital excitation with consistently large CI coefficients (ca. 0.70). Notably, the **E**, **KE**, and **KK** forms generally retain relatively high oscillator strengths, whereas the fully keto **K** forms show substantially reduced intensities. Overall, the results demonstrate that the tautomeric process strongly modulates both the excitation energy and oscillator strength, providing an effective mechanism for tuning the absorption properties of both stator and proton-crane systems.

Tautomerization narrows the HOMO–LUMO gap in the stator compounds, with keto forms showing smaller gaps than their enol counterparts, consistent with the computed bathochromic shifts (Figure 3). This is accompanied by redistribution of electron density within the frontier orbitals, indicating altered conjugation and donor–acceptor characteristics—a modulation of frontier orbital energies that consistently accounts for the observed shifts, establishing tautomerization as an effective means of tuning the electronic and optical properties of these systems.

A clear narrowing of the HOMO–LUMO gap upon conversion from the enol to the keto forms, similar to that observed for the stator compounds, is also seen for both proton-crane systems shown in Figure 4: **HQBT**, the experimentally validated proton crane, and **HCBT**, both serving as positive references. For HQBT, the gap decreases from 6.280 eV (E) to 5.639 eV (K), while for HCBT it decreases from 6.035 to 5.334 eV. This gap reduction, accompanied by changes in frontier orbital distributions, is consistent with the red-shifted electronic transitions predicted for the keto tautomers and highlights the role of proton transfer in tuning the electronic and optical properties of these systems. For comparison, the corresponding orbital analysis for an underperforming (negative-example) proton crane (**HBOBT**) is provided in Figure S3. Across all three systems, the HOMO–LUMO gap narrows along the **E** → **KE** → **KK** → **K** sequence, with **HBOBT** showing the largest overall reduction (6.494 → 5.446 eV) compared to **HQBT** (6.280 → 5.639 eV) and **HCBT** (6.035 → 5.334 eV). However, in **HQBT** and **HCBT**, this narrowing culminates in a distinct LUMO stabilization at the final **K** step, whereas in **HBOBT**, the gap closes primarily through HOMO destabilization throughout, without this late-stage LUMO contribution.

It should be emphasized that the discussion of the possible proton-crane action of these compounds is based on the assumption that the level of theory used correctly describes the PES in the ground state. This has been previously confirmed for **HQBT**. The remaining compounds have never been studied experimentally. However, data are available for the benzoxazole (BO) analogs of **HQOBT** and **HBTDBT**, namely **HQOBO** and **HBTDBO** [77,78]. The results indicate that the presence of electron-withdrawing stators, such as thiadiazole and pyrazine, suppresses the ESIPT process, and only a single emission coming from the excited enol tautomer is observed in dichloromethane. This is one possible interpretation. The other is that the excited-state **KE** form, once formed, twists and reaches the ground state in the conical intersection region without emission. This highlights the importance of further experimental studies.

## 4. Conclusions

The effect of various OH-containing heterocyclic stators, retaining the tautomeric structural motif of quinoline-7-ol, was investigated using benzothiazole as a rotor. The results indicate that when quinoxalin-6-ol, quinazolin-7-ol, benzo[c][1,2,5]oxadiazol-5-ol, benzo[c][1,2,5]thiadiazol-5-ol, benzo[d]oxazol-5-ol, or benzo[d]thiazol-5-ol are used as stators, long-range intramolecular proton transfer is not possible in the ground state, in either toluene or acetonitrile. In toluene, the cinnolin-7-ol-based proton crane **HCBT** shows a very promising potential energy surface, with improvement relative to previously studied **HQBT**—a substantial finding, given the reduced efficiency of the latter in non-polar media. The potential energy surface of **HCBT** suggests that clean switching from **E** to **K** could be possible.

## Figures and Tables

**Table 1:** Relative ground-state stabilities and the long-wavelength absorption bands of the tautomers of the compounds from Scheme 1 in toluene. The relative energies in acetonitrile are given in brackets.

Compound	E				K			
	ΔE	ΔG	μ	λ_max_	ΔE	ΔG	μ	λ_max_
	[kcal/mol]		[D]	[nm]	[kcal/mol]		[D]	[nm]
**HQ**	0.0	0.0	1.5	301	11 (6.8)	12 (7.2)	10.0	430
**HQO**	0.0	0.0	1.2	340	15 (11)	15 (11)	8.4	453
**HQZ** *	0.0	0.0	2.8	313 ** 289	11 (6.9)	11 (7.5)	6.7	395
**HC**	0.0	0.0	3.9	404 ** 321	6.1 (4.5)	7.1 (5.4)	7.2	462
**HBOD**	0.0	0.0	4.7	317	20 (19)	20 (18)	3.5	408 ** 372
**HBTD**	0.0	0.0	2.0	339	18 (15)	17 (14)	6.7	401
**HBO**	0.0	0.0	0.2	269	35 (25)	34 (25)	13.7	498
**HBT**	0.0	0.0	0.4	285	31 (22)	31 (22)	13.4	575

* The relative energy/Gibbs free energy of the third, N_3_H, tautomer is 13/13 kcal/mol in toluene and 7.3/8.0 in acetonitrile; ** dark state.

**Table 2:** HOMA indices and NBO charges (in brackets) of the rings of the tautomeric forms in the ground-states in toluene.

Compound	E		K	
	OH Ring	N Ring	O Ring	NH Ring
**HQ**	0.809 (0.134)	0.869 (−0.107)	0.224 (−0.297)	0.766 (0.369)
**HQO**	0.815 (0.332)	0.881 (−0.136)	0.028 (0.005)	0.690 (0.298)
**HQZ**	0.832 (0.165)	0.911 (−0.150)	0.145 (−0.384)	0.764 (0.414)
**HC**	0.783 (0.177)	0.923 (−0.154)	0.104 (−0.313)	0.708 (0.412)
**HBOD**	0.525 (0.281)	0.635 (−0.167)	−0.200 (−0.004)	0.248 (0.251)
**HBTD**	0.557 (0.216)	- * (−0.093)	−0.144 (0.021)	- * (0.276)
**HBO**	0.984 (0.373)	0.282 (−0.058)	0.597 (−0.067)	0.400 (0.508)
**HBT**	0.969 (−0.336)	0.698 (−0.132)	0.602 (−0.698)	0.839 (0.510)

* The parametrization for the N–S bond is not available.

**Table 3:** Relative ground-state stabilities of the tautomers of the compounds from Scheme 3 in toluene. The relative energies in acetonitrile are given in brackets.

	E			KE			KK			K		
	ΔE	ΔG	μ	ΔE	ΔG	μ	ΔE	ΔG	μ	ΔE	ΔG	μ
	[kcal/mol]		[D]	[kcal/mol]		[D]	[kcal/mol]		[D]	[kcal/mol]		[D]
**HQBT**	0.0	0.0	2.9	5.5 (4.5)	5.1 (4.3)	2.9	6.2 (4.3)	6.4 (4.6)	5.9	6.4 (3.1)	5.9 (2.9)	8.8
**HQOBT**	0.0	0.0	2.4	5.1 (4.1)	5.3 (4.2)	4.9	6.2 (4.2)	7.1 (5.0)	6.6	9.8 (6.8)	11 (7.8)	7.0
**HQZBT**	0.0	0.0	0.8	4.5 (3.5)	5.1 (3.8)	3.7	5.2 (3.4)	6.6 (4.5)	4.4	7.0 (4.4)	8.4 (5.3)	5.1
**HCBT**	0.0	0.0	2.6	4.2 (3.3)	4.2 (3.3)	0.7	4.0 (2.9)	4.6 (3.5)	2.8	2.74 (1.25)	3.0 (1.09)	6.6
**HBODBT**	0.0	0.0	3.4	2.27 (1.04)	2.33 (1.14)	5.7	2.88 (1.39)	3.2 (1.71)	4.5	19 (17)	18 (16)	3.3
**HBTDBT**	0.0	0.0	1.3	3.4 (2.33)	3.2 (2.20)	4.4	3.8 (2.12)	4.0 (2.46)	5.1	15 (12)	14 (11)	5.4
**HBOBT**	0.0	0.0	2.0	10 (8.7)	9.0 (8.2)	4.0	12 (9.3)	12 (9.6)	6.4	26 (20)	26 (20)	11.0
**HBTBT**	0.0	0.0	2.3	8.9 (7.7)	7.1 (7.0)	3.8	10 (7.9)	11 (8.4)	6.5	21 (15)	20 (15)	10.8

## Data Availability

The original contributions presented in this study are included in the article/Supplementary Materials. Further inquiries can be directed to the corresponding author.

## Supplementary Materials

The following supporting information can be downloaded at: https://www.mdpi.com/article/10.3390/mi17091084/s1, Table S1. NBO natural charges on the heteroatoms in the structures shown in Scheme 1, calculated in toluene. Table S2. Calculated vertical excitation wavelengths (λ) and oscillator strengths (*f*) of the dominant S_1_ (or S_2_, where S_1_ is optically dark) transition for the enol (**E**) and keto (**KE**, **KK**, **K**) tautomeric forms of the stator and proton-crane compounds, along with the corresponding dominant orbital transition and its CI expansion coefficient. Figure S1. Predicted absorption spectra of the tautomers of the compounds, shown in Scheme 1, in toluene. Figure S2. Ground-state PESs of the compounds from Scheme 3 in toluene and acetonitrile. The numerical values for the relative stabilities of the tautomers are collected in Table 3. Figure S3. Frontier molecular orbital energy diagrams and spatial distributions of the HOMO and LUMO for the **E**, **KE**, **KK**, and **K** tautomeric forms of the proton-crane compound **HBOBT**. The corresponding HOMO and LUMO energies and HOMO–LUMO gaps (ΔE, eV) are indicated for each tautomer. Purple and green isosurfaces represent opposite phases of the molecular orbitals.
