# Supplementary material for: Improving Long-Range Intramolecular Proton Transfer—Further Molecular Design Using the Successful Molecular Switch 8-(Benzo[d]thiazol-2-yl)quinolin-7-ol (HQBT) as a Structural Model
Source: Micromachines (Basel). 2026 Sep 15;17(9):1084. doi: 10.3390/mi17091084 (PMC13609328; doi:10.3390/mi17091084)
Supplement: Supplementary file 1 [file micromachines-17-01084-s001.zip › micromachines-4504579-supplementary.pdf]

Supporting Information

Improving long-range intramolecular proton transfer – further molecular design using the successful molecular switch 8-(benzo[d]thiazol-2-yl)quinolin-7-ol (HQBT) as a structural model

Daniela Nedeltcheva-Antonova,[a,b] Nikoleta Kircheva,[a,c,d] Silvia Angelova,[c,d] and Liudmil Antonov [a,c,*]

a) Institute of Electronics, Bulgarian Academy of Sciences, Sofia 1784, Bulgaria

b) Institute of Organic Chemistry with Centre of Phytochemistry, Bulgarian Academy of Sciences, Sofia 1113, Bulgaria

c) Institute of Optical Materials and Technologies "Acad. J. Malinowski", Bulgarian Academy of Sciences, Sofia 1113, Bulgaria

d) University Centre on Tautomeric Research and Education in Science and Technology (ERA Chair UCTREST), University of Plovdiv, Plovdiv 4000, Bulgaria

* corresponding author: lantonov@tautomer.eu

Table S1. NBO natural charges on the heteroatoms in the structures shown in Scheme 1, calculated in toluene.

Compound	E				K			
	O (PD)	N (PA)	N	O/S	O (PA)	N (PD)	N	O/S
**HQ**	-0.655	-0.421	-	-	-0.642	-0.475	-	-
**HQO**	-0.651	-0.379	-0.361	-	-0.612	-0.469	-0.341	-
**HQZ**	-0.647	-0.441	-0.466	-	-0.618	-0.502	-0.487	-
**HC**	-0.650	-0.198	-0.197	-	-0.617	-0.305	-0.163	-
**HBOD**	-0.646	-0.099	-0.068	-0.115	-0.568	-0.288	-0.022	-0.150
**HBTD**	-0.650	-0.595	-0.574	0.868	-0.596	-0.702	-0.512	0.748
**HBO**	-0.660	-0.453	-	-0.401	-0.714	-0.470	-	-0.324
**HBT**	-0.658	-0.418	-	0.363	-0.709	-0.443	-	0.559

HQ

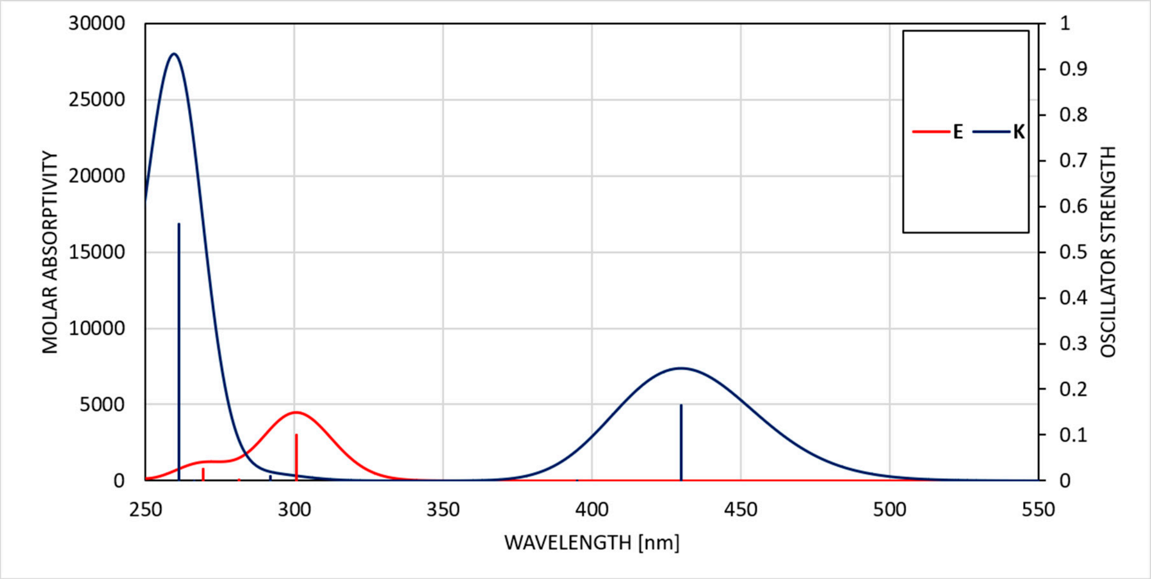

30000
25000
20000
15000
10000
5000
0
1
0.9
0.8
0.7
0.6
0.5
0.4
0.3
0.2
0.1
0
250
300
350
400
450
500
550
MOLAR ABSORPTIVITY
OSCILLATOR STRENGTH
WAVELENGTH [nm]
E
K

HQO

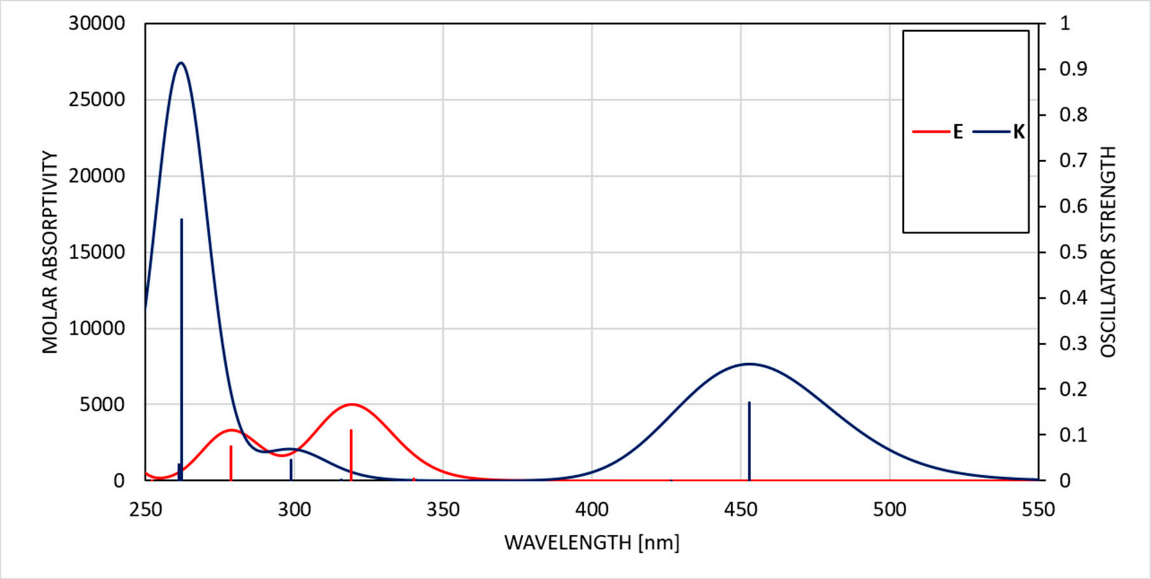

30000
25000
20000
15000
10000
5000
0
1
0.9
0.8
0.7
0.6
0.5
0.4
0.3
0.2
0.1
0
250
300
350
400
450
500
550
MOLAR ABSORPTIVITY
OSCILLATOR STRENGTH
WAVELENGTH [nm]
E
K

HQZ

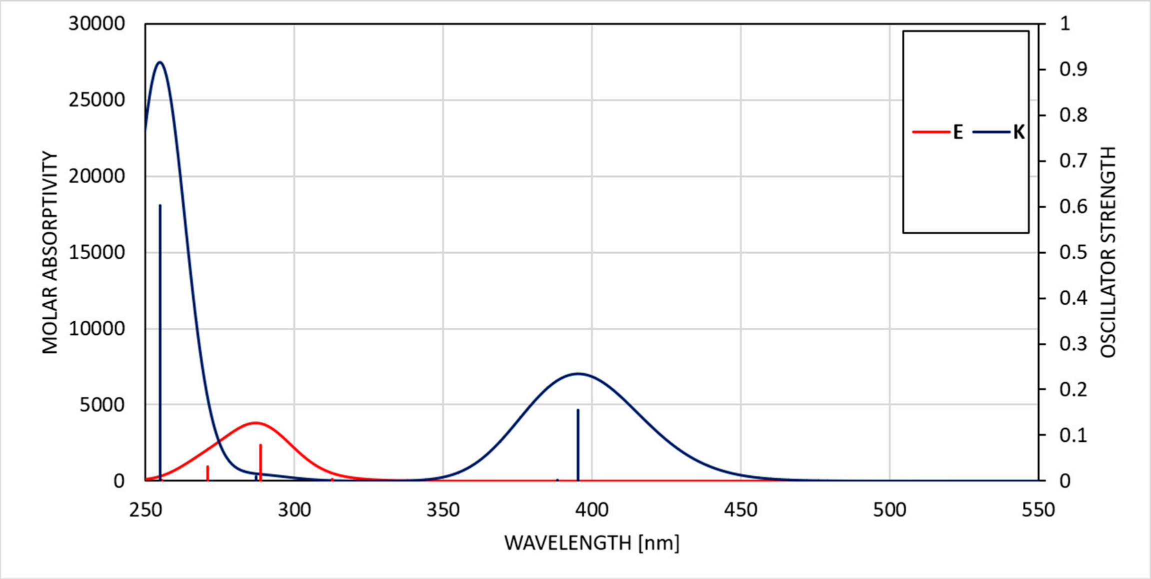

MOLAR ABSORPTIVITY
OSCILLATOR STRENGTH
WAVELENGTH [nm]
E
K
30000
25000
20000
15000
10000
5000
0
1
0.9
0.8
0.7
0.6
0.5
0.4
0.3
0.2
0.1
0
250
300
350
400
450
500
550

HC

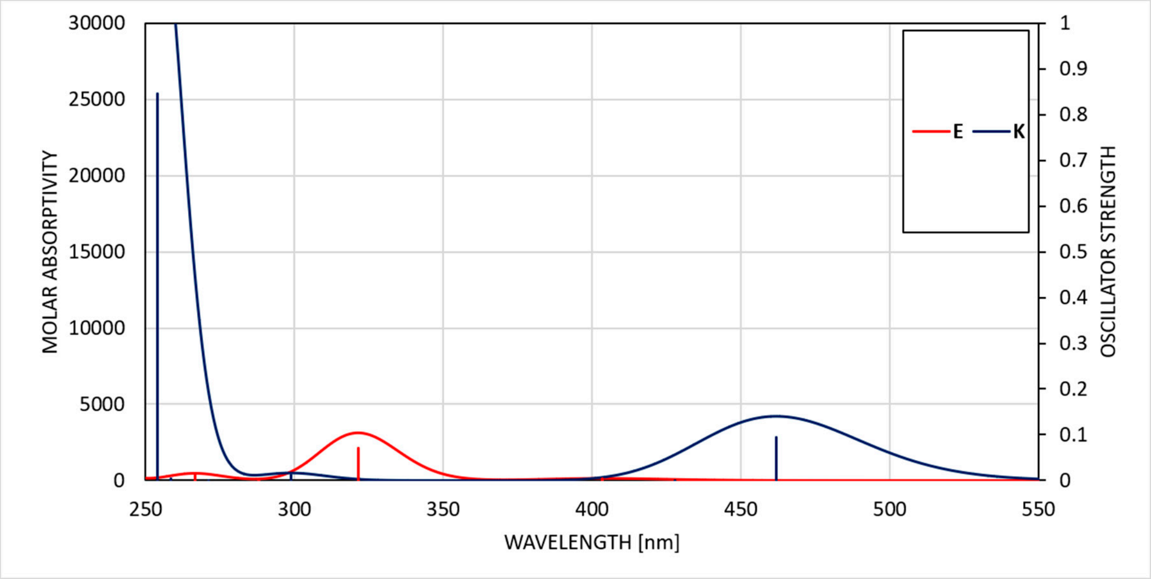

MOLAR ABSORPTIVITY
OSCILLATOR STRENGTH
WAVELENGTH [nm]
E
K
30000
25000
20000
15000
10000
5000
0
1
0.9
0.8
0.7
0.6
0.5
0.4
0.3
0.2
0.1
0
250
300
350
400
450
500
550

HBOD

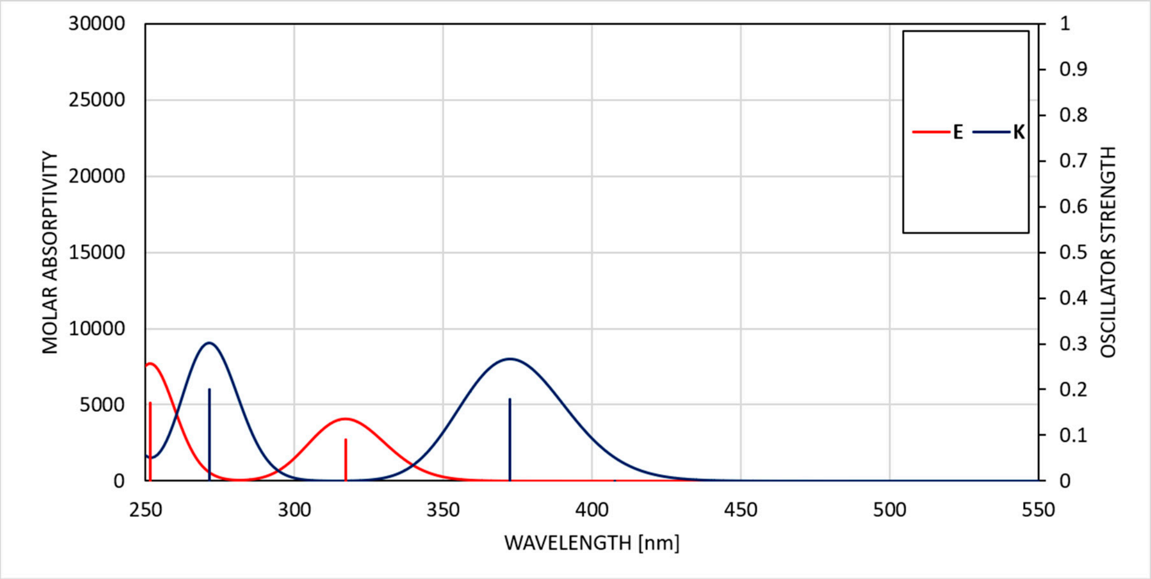
30000
25000
20000
15000
10000
5000
0
MOLAR ABSORPTIVITY
1
0.9
0.8
0.7
0.6
0.5
0.4
0.3
0.2
0.1
0
OSCILLATOR STRENGTH
250
300
350
400
450
500
550
WAVELENGTH [nm]
E
K

HBTD

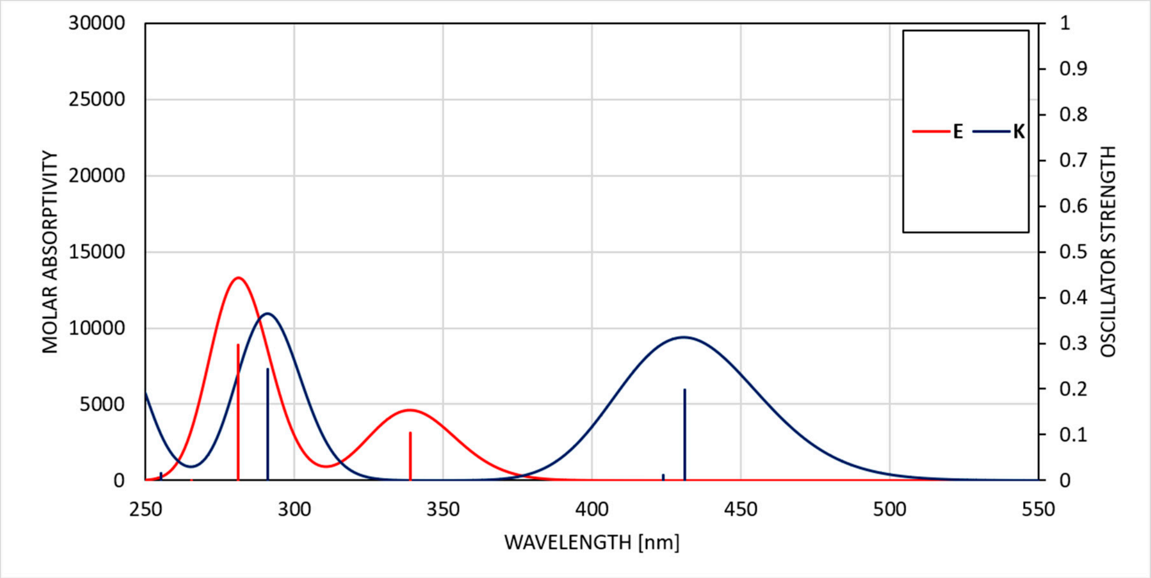
30000
25000
20000
15000
10000
5000
0
MOLAR ABSORPTIVITY
1
0.9
0.8
0.7
0.6
0.5
0.4
0.3
0.2
0.1
0
OSCILLATOR STRENGTH
250
300
350
400
450
500
550
WAVELENGTH [nm]
E
K

HBO

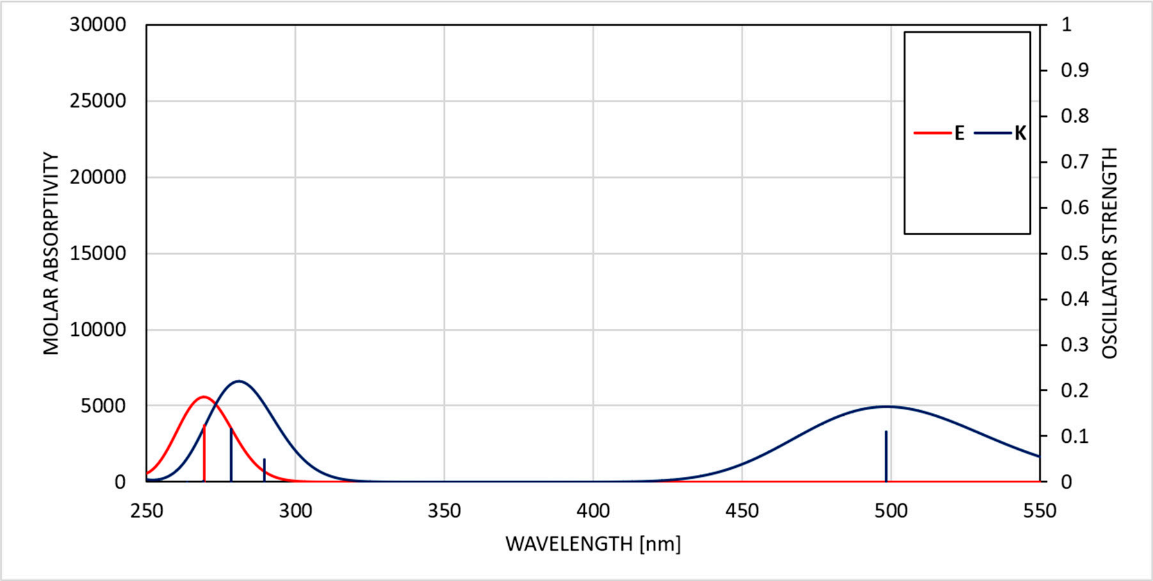

HBT

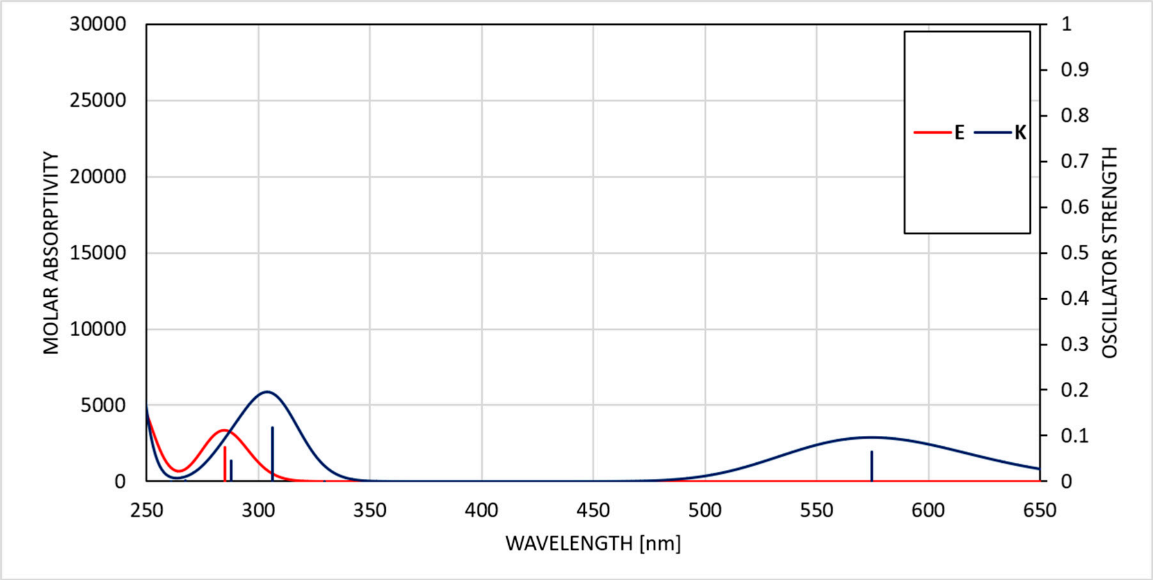

Figure S1. Predicted absorption spectra of the tautomers of the compounds, shown in Scheme 1, in toluene.

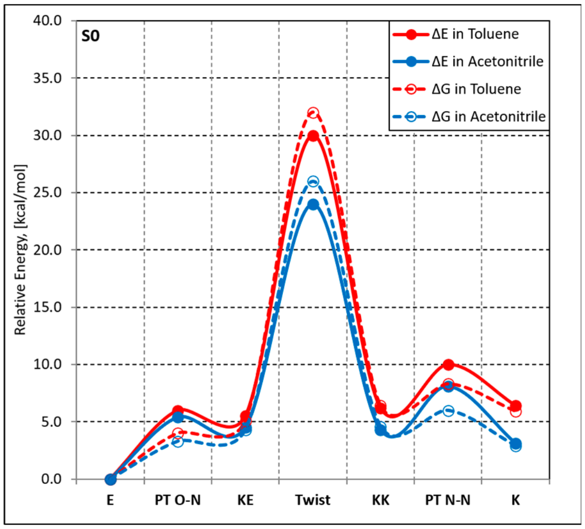

HQBT

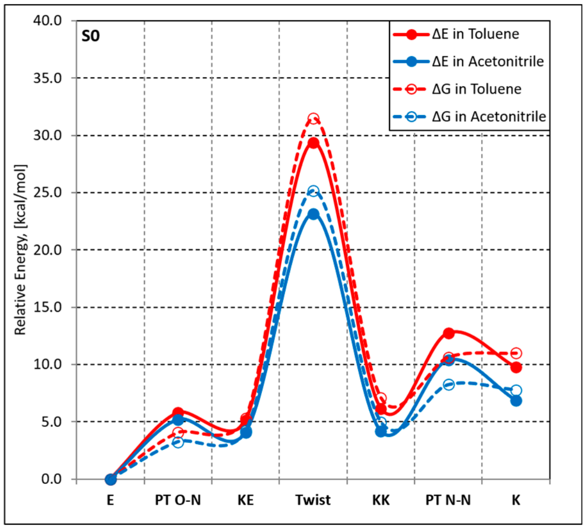

HQOBT

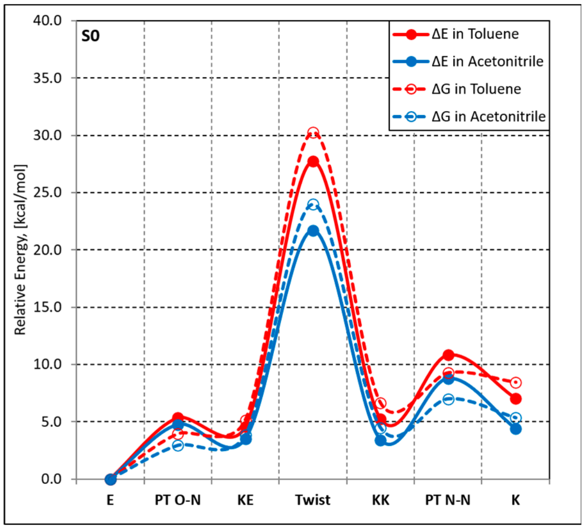

HQZBT

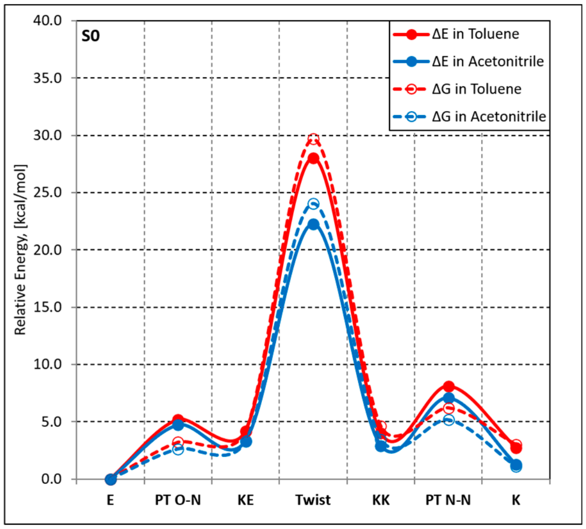

HCBT

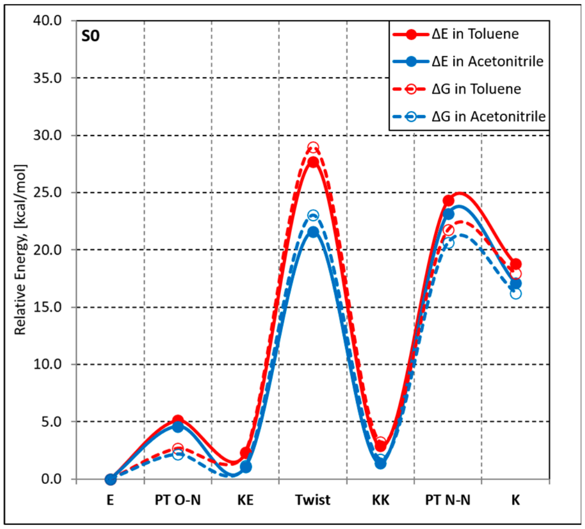

HBODBT

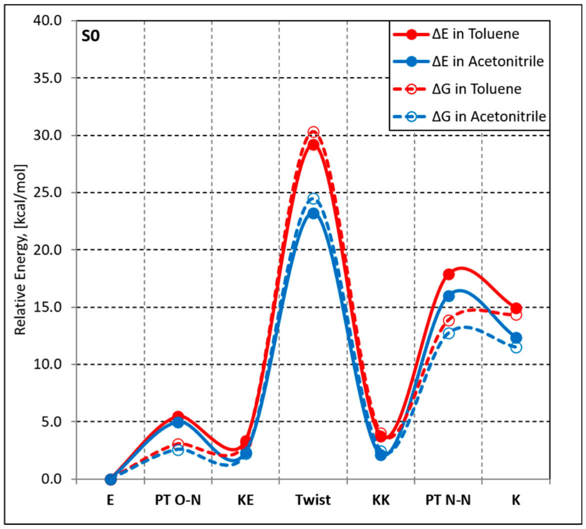

HBTDBT

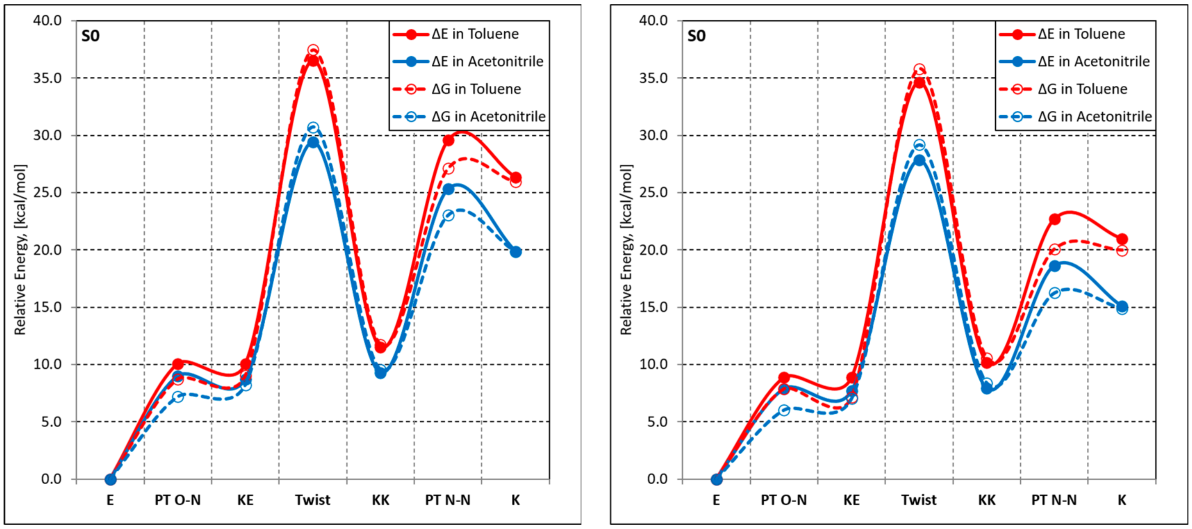

HBOBT **HBTBT**

Figure S2. Ground state PESs of the compounds from Scheme 3 in toluene and acetonitrile. The numerical values for the relative stabilities of the tautomers are collected in Table 3.

Table S2. Calculated vertical excitation wavelengths (λ) and oscillator strengths (*f*) of the dominant S_1 (or S_2, where S_1 is optically dark) transition for the enol (**E**) and keto (**KE**, **KK**, **K**) tautomeric forms of the stator and proton-crane compounds, along with the corresponding dominant orbital transition and its CI expansion coefficient.

Compound / Tautomer	State: transition	coefficient	λ [nm]	*f*
		Stators		
**HQ / E**	S1: 38 -> 39	0.67388	300.74	0.0989
**HQ / K**	S1: 38 -> 39	0.69039	429.88	0.1637
**HQO / E**	S1 – dark state<br>S2: 38 -> 39	0.68946	319.16	0.1100
**HQO / K**	S1: 38 -> 39	0.68780	452.90	0.1697
**HQZ / E**	S1 – dark state<br>S2: 38 -> 39	0.67235	288.67	0.0774
**HQZ / K**	S1: 38 -> 39	0.67879	395.38	0.1547
**HC / E**	S1: 38 -> 39<br>(nearly dark)	0.70233	403.52	0.0031
**HC / K**	S1: 38 -> 39	0.69198	461.82	0.0935
**HBOD / E**	S1: 35 -> 36	0.69997	317.10	0.0904
**HBOD / K**	S1 – dark state<br>S2: 35 -> 36	0.67036	372.39	0.1774
**HBTD / E**	S1: 39 -> 40	0.69430	338.77	0.1027
**HBTD / K**	S1: 39 -> 40	0.66784	431.08	0.1985
**HBO / E**	S1: 35 -> 36	0.67771	269.18	0.1241
**HBO / K**	S1: 35 -> 36	0.70236	498.41	0.1096
**HBT / E**	S1: 39 -> 40	0.68066	284.86	0.0748
**HBT / K**	S1: 39 -> 40	0.70361	574.59	0.0642
		Proton cranes		
**HQBT / E**	S1: 72 -> 73	0.69908	358.64	0.4608
**HQBT / KE**	S1: 72 -> 73	0.70210	390.18	0.4840
**HQBT / KK**	S1: 72 -> 73	0.70094	395.37	0.4363
**HQBT / K**	S1: 72 -> 73	0.69687	446.81	0.0803
**HCBT / E**	S1 – dark state<br>S2: 72 -> 73	0.69851	390.22	0.3204
**HCBT / KE**	S1: 72 -> 73	0.70032	411.86	0.3364
**HCBT / KK**	S1: 72 -> 73	0.69946	422.96	0.2494
**HCBT / K**	S1: 72 -> 73	0.70024	513.34	0.0418
**HBOBT / E**	S1: 69 -> 70	0.69872	342.93	0.5236
**HBOBT / KE**	S1: 69 -> 70	0.70475	411.38	0.4025
**HBOBT / KK**	S1: 69 -> 70	0.70329	417.83	0.4272
**HBOBT / K**	S1: 69 -> 70	0.69843	477.29	0.1313

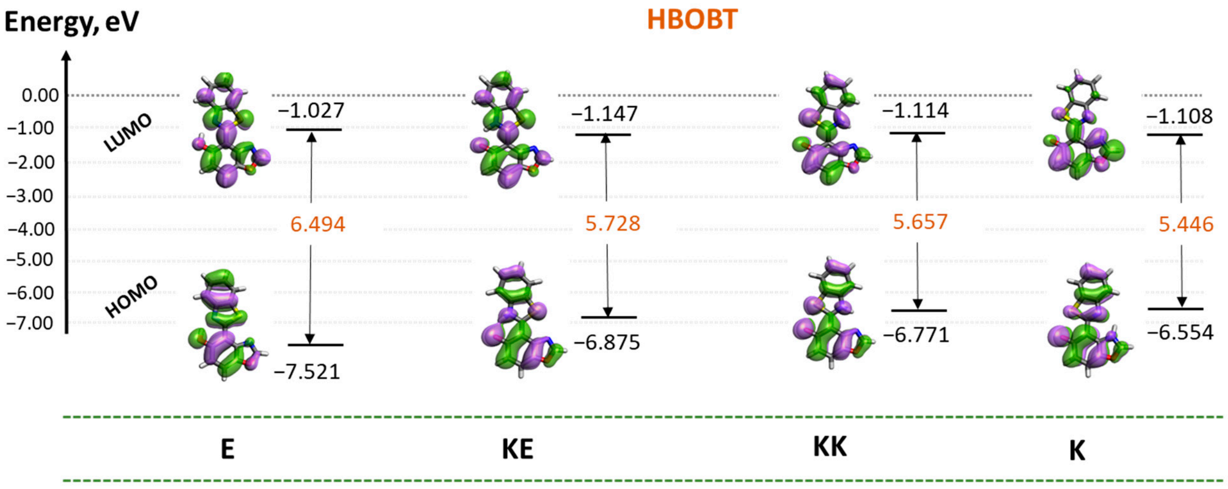

Figure S3. Frontier molecular orbital energy diagrams and spatial distributions of the HOMO and LUMO for the **E**, **KE**, **KK**, and **K** tautomeric forms of the proton-crane compound **HBOBT**. The corresponding HOMO and LUMO energies and HOMO–LUMO gaps (ΔE, eV) are indicated for each tautomer. Purple and green isosurfaces represent opposite phases of the molecular orbitals.
